# A comprehensive and universal approach for embryo testing in patients with different genetic disorders

**DOI:** 10.1002/ctm2.490

**Published:** 2021-07-08

**Authors:** Shuo Zhang, Caixia Lei, Junping Wu, Min Xiao, Jing Zhou, Saijuan Zhu, Jing Fu, Daru Lu, Xiaoxi Sun, Congjian Xu

**Affiliations:** ^1^ Shanghai Ji Ai Genetics & IVF Institute, Obstetrics and Gynecology Hospital Fudan University Shanghai China; ^2^ Key Laboratory of Female Reproductive Endocrine Related Diseases, Obstetrics and Gynecology Hospital Fudan University Shanghai China; ^3^ State Key Laboratory of Genetic Engineering, School of Life Science Fudan University Shanghai China; ^4^ NHC Key Laboratory of Birth Defects and Reproductive Health, Chongqing Key Laboratory of Birth Defects and Reproductive Health, Chongqing Population and Family Planning Science and Technology Research Institute Chongqing China

**Keywords:** chromosomal rearrangements, chromosome aneuploidies, family haplotype linkage analysis, monogenic diseases, preimplantation genetic testing

## Abstract

**Background:**

*In vitro* fertilization (IVF) with preimplantation genetic testing (PGT) has markedly improved clinical pregnancy outcomes for carriers of gene mutations or chromosomal structural rearrangements by the selection of embryos free of disease‐causing genes and chromosome abnormalities. However, for detecting whole or segmental chromosome aneuploidies, gene variants or balanced chromosome rearrangements in the same embryo require separate procedures, and none of the existing detection platforms is universal for all patients with different genetic disorders.

**Methods:**

Here, we report a cost‐effective, family‐based haplotype phasing approach that can simultaneously evaluate multiple genetic variants, including monogenic disorders, aneuploidy, and balanced chromosome rearrangements in the same embryo with a single test. A total of 12 monogenic diseases carrier couples and either of them carried chromosomal rearrangements were enrolled simultaneously in this present study. Genome‐wide genotyping was performed with single‐nucleotide polymorphism (SNP)‐array, and aneuploidies were analyzed through SNP allele frequency and Log R ratio. Parental haplotypes were phased by an available genotype from a close relative, and the embryonic genome‐wide haplotypes were determined through family haplotype linkage analysis (FHLA). Disease‐causing genes and chromosomal rearrangements were detected by haplotypes located within the 2 Mb region covering the targeted genes or breakpoint regions.

**Results:**

Twelve blastocysts were thawed, and then transferred into the uterus of female patients. Nine pregnancies had reached the second trimester and five healthy babies have been born. Fetus validation results, performed with the amniotic fluid or umbilical cord blood samples, were consistent with those at the blastocyst stage diagnosed by PGT.

**Conclusions:**

We demonstrate that SNP‐based FHLA enables the accurate genetic detection of a wide spectrum of monogenic diseases and chromosome abnormalities in embryos, preventing the transfer of parental genetic abnormalities to the fetus. This method can be implemented as a universal platform for embryo testing in patients with different genetic disorders.

## Graphical abstract

1. We report a cost‐effective, comprehensive, and universal platform for embryo testing in patients with different genetic disorders.

2. SNP‐based FHLA enables the accurate genetic detection for a wide spectrum of monogenic diseases and chromosome rearrangements in embryos.

3. This proposed strategy may markedly improve the precision of embryo testing and prevent the birth of affected fetuses.

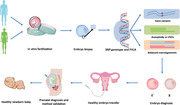

**Abbreviations**
ADO: allele dropout
CCS: comprehensive chromosome screening
CGH: array‐comparative genomic hybridization
CNV: copy number variation
FHLA: family haplotype linkage analysis
FISH: fluorescent *in‐situ* hybridization
hCG: human chorionic gonadotrophin
IVF: *in vitro* fertilization
MaReCs: mapping allele with resolved carrier status
MARSALA: mutated allele revealed by sequencing with aneuploidy and linkage analyses
PCR: polymerase chain reaction
PGT: preimplantation genetic testing
PGT‐A: preimplantation genetic testing for aneuploidy
PGT‐M: preimplantation genetic testing for monogenic disease
PGT‐SR: preimplantation genetic testing for structural rearrangement
SNP: single‐nucleotide polymorphism
SNV: single‐nucleotide variant
STR: short tandem repeats
WGA: whole‐genome amplification

## 1 INTRODUCTION

Genetic diseases can be life threatening, often manifesting early in life. Till now, there are an estimated 6000‐8000 kinds of rare diseases, the main cause of which is the single gene variants.^1^ Although the individual diseases are rare, the total number of affected people exceeds 200 million worldwide and almost all of the rare diseases exert a large impact on healthcare throughout the world.^1^, ^2^ In spite of the identification of genetic variants for many rare diseases, treatments exist for only about 6% of these diseases, of which fewer than 1% are curative,^3^ therefore preventing the birth of affected fetuses is crucial. In addition, chromosomal structural rearrangements have long been known to significantly impact fertility as they cause chromosomal imbalances and aneuploidy in gametes, and are considered to be a high‐risk factor for miscarriages and congenital malformations.^4^, ^5^ Balanced translocations and inversions are the most common types of rearrangements, a large cohort study in Denmark showed that the prevalence of balanced translocations is about 2.66/1000 in newborns,^6^ and the probability increases up to 4.08% in couples with recurrent miscarriages.^7^

Preimplantation genetic testing (PGT) has significantly improved the clinical diagnosis rate and pregnancy rate for carriers of chromosomal structural rearrangements or monogenic diseases, especially with the growing use of comprehensive chromosome screening (CCS) techniques, such as next‐generation sequencing (NGS), array‐comparative genomic hybridization(CGH), and single‐nucleotide polymorphism (SNP)‐array, which can detect chromosomal imbalances and screen for aneuploidy simultaneously across the genome.^8^, ^9^, ^10^, ^11^ For carriers of monogenic diseases, sequencing has been used to directly detect mutations in a single embryo cell, in the whole‐genome amplification (WGA) products of biopsied trophectoderm (TE) cells or polar body.^12^, ^13^, ^14^ However, every WGA methods can result in false negative or positive single‐nucleotide variants (SNVs) owing to amplification preference of the primers and/or allele dropout (ADO), leading to misdiagnosis.^15^, ^16^ Family‐based linkage analysis has now become a popularized method by testing short tandem repeats (STRs) or SNPs to indirectly determine variants by haplotype,^17^, ^18^, ^19^, ^20^, ^21^, ^22^ and some of these researches have also confirmed that gene variants and chromosome aneuploidies can be detected simultaneously.^18^, ^19^, ^20^, ^21^, ^22^ Massively parallel sequencing, like MARSALA, enables direct genotyping and haplotype analysis.^18^, ^19^ SNP‐array genotype combined with karyomapping or haplarithmisis analysis provides a genome‐wide haplotyping for monogenic diseases.^21^, ^22^ However, the availability of an affected proband was required in these reported methods, it was difficult to conduct linkage analysis in families that did not retain the proband's DNA or tissue. For carriers of chromosome rearrangements, the procedure was performed initially using fluorescent *in‐situ* hybridization (FISH) to test diploid embryos, but some randomized controlled trials demonstrated that FISH technology was inefficient and did not increase the delivery rates following the detection of the limited chromosome number.^23^, ^24^ More recently, CCS techniques were introduced for such analyses.^8^, ^9^, ^10^, ^11^ Fortunately, some current methods not only detect chromosome imbalances, but they can also further distinguish embryos with balanced rearrangement karyotypes from those with normal karyotypes.^25^, ^27^ This helps avoid the passing on chromosome rearrangements to their next generation, which is of great importance. However, these methods cannot detect chromosome aneuploidies and chromosome rearrangements simultaneously in an embryo, and separate procedures had to be performed. In addition, these methods also require breakpoint identification of rearrangements to a single base by sequencing, which is not suitable for routine clinical applications, and it is not universal for carriers with different rearrangements. In contrast, our previous research proved that aneuploidies and chromosomal rearrangements can be detected simultaneously by SNP‐array‐based haplotype analysis.^28^, ^29^ SNP‐array also yields good results while detecting triploidy and uniparental disomy.^4^, ^10^ In addition, for couples with recurrent miscarriages or advanced maternal age, CCS techniques are now widely used to detect aneuploidies and select diploid embryos for transfer.^30^, ^32^

In this study, we report a new cost‐effective, family‐based haplotype phasing approach that can simultaneously evaluate multiple variants, including monogenic disorders, aneuploidy, and balanced chromosome rearrangements in the same embryo with a single test, thus providing the scope of avoiding the inheritance of genetic variants to the next generations. Also, this is a universal technique for PGT with different genetic disorders, such as various monogenic diseases and chromosomal rearrangements. In addition, for monogenic diseases, an affected family member, such as a proband, is not required in the proposed method.

## 2 METHODS AND MATERIALS

### 2.1 Study design

Twelve families that sought treatment for assisted reproductive treatment were collected at the Shanghai Ji Ai Genetics & IVF Institute between June 2018 and May 2020. One individual in each couple was a carrier of apparently chromosomal rearrangements, and the spouses of all carriers had normal karyotypes and each couple was the carriers of the same disease‐causing gene. Peripheral blood (20 mL) was drawn from each carrier's parents to confirm the origins of their variants. Karyotype analyses were performed with conventional G‐banding, and gene variants were validated by Sanger sequencing, all of these variants were inherited from one parent in this study. All of the families enrolled had a history of infertility or abnormal pregnancies. Each family was required to sign an informed consent form before the PGT cycle was started and the study protocol was approved by the Ethics Committee for Human Subject research of the Obstetrics and Gynecology Hospital, Fudan University.

### 2.2 G‐band karyotyping and Sanger sequencing

Culturing, harvesting, and metaphase preparation of the peripheral/umbilical cord blood and amniotic fluid sample were carried out as previously described.^33^ In general, at least 20 mitoses for amniotic fluid and 100 mitoses for peripheral blood were analyzed, and nomenclature was performed according to the International System for Human Cytogenomic Nomenclature (ISCN, 2016). In addition, genomic DNA of blood and amniotic fluid was extracted using a DNeasy kit (QIAGEN GmbH, Hilden, Germany) as described in the manufacturer's protocol. Subsequently, mutations of disease‐causing gene were amplified with primers for Sanger sequencing. Polymerase chain reaction (PCR) was set up, including 0.2 μM primers, 200 μM dNTP, 1.25 U Taq polymerase, and 50 ng DNA template. A preheating step was carried out at 94°C for 3 minutes, followed by 35 cycles of denaturation (94°C for 30 seconds), annealing (60°C for 30 seconds), and extension (72°C for 30 seconds). A final extension step was carried out at 72°C for 5 minutes.

### 2.3 Blastocyst biopsy and WGA

The standard techniques were used for *in vitro* fertilization (IVF). Briefly, retrieved MII oocytes were produced using intracytoplasmic sperm injection, and then were cultured to develop to the blastocyst stage. The criteria for grading blastocyst were according to the recommendation by Schoolcraft et al.^34^ Blastocyst biopsy was performed, and three to five TE cells were collected and immediately transferred to PCR tubes with phosphate buffer saline for WGA by multiple displacement amplification (MDA) technology. Isothermal DNA amplification with phi 29 DNA polymerase using a Repli‐g Single Cell kit (QIAGEN GmbH, Hilden, Germany) was performed as the manufacturer's protocol. The isothermal reaction was incubated at 30°C for 8 hours and the amplification was terminated by maintaining 65°C for 3 minutes.

### 2.4 Haplotype phasing

The WGA products and blood DNA of family members were processed with SNP‐array according to the manufacturer's instructions, which were then scanned using iScan Bead Array Reader (Illumina, San Diego, CA, USA). SNP calling was performed and informative SNPs were defined. The criterion for informative SNP was that these SNPs should be heterozygous in the carrier and homozygous in his/her partner, meanwhile being homozygous in the carrier's family members when used as references for haplotyping. When the carrier's child or unbalanced embryo was used as a reference, the informative SNPs were defined as heterozygous in the carrier and homozygous in his/her partner. Usually, the regions within −2 Mbp flanking disease‐causing genes or the breakpoints were chosen to avoid misinterpretation from possible recombination events that might have occurred during meiosis. Therefore, the whole genome in our research was divided into a large numbers of 2 Mbp segments, which were called windows. Analytical performance was assessed by comparing the number of total SNPs and effective SNPs in each window. The number of total SNPs was used for calculating copy number variations (CNVs). The number of informative SNPs was crucial to linkage analysis and was used to determine the accuracy of haplotype classification directly. This was limited by each specific family and associated with the distribution of SNP allele frequency. For each family, SNPs with high minor allele frequency were more likely to act as the informative SNPs.

Based on informative SNPs and haplotype phasing principles, we developed our pipeline, which was programmed in Practical Extraction and Reporting Language (Perl), and was capable of obtaining the clear haplotypes of each family member. When all the family samples including blood and embryos were detected in a single test, the raw scanning data would be imported into the pipeline and then produce the accurate haplotype and chromosomal aneuploidy results throughout the genome, in which the positions of the disease‐causing gene and the rearrangement breakpoints were critically focused on. Either parent could be used for haplotyping, it may be noted that the analysis process will be opposite between the parent with the variant and the normal parent. Generally, at least no less than two informative SNPs were required for haplotype inference, one window with 2 Mb had sufficient informative SNPs. Assuming that the evidence was clear out or some regions in genome had almost no SNPs, the haplotypes of surrounding windows or regions would be helpful.

### 2.5 Testing for aneuploidy, disease‐causing gene, and chromosome rearrangement

For analysis of aneuploidy or CNVs, the microarray scanning results were processed using the B allele frequency and Log R ratio, and the core algorithm was according to the cnvPartition as reported.^35^, ^36^ For chromosome rearrangements, the molecular karyotype of an unbalanced embryo could help to pinpoint the relatively accurate breakpoints positions, and informative SNPs of 2 Mbp region around the breakpoints were focused upon to establish haplotypes. When no unbalanced embryo was identified, the breakpoints from the peripheral blood karyotype were used, and the range used for linkage analysis extended to 5‐10 Mb. This information was used to determine which one of the carrier's two haplotypes were linked to the rearranged chromosome or to the normal chromosome. If there are two haplotypes of rearranged chromosome in breakpoint regions, the embryo will be diagnosed as embryo with rearrangements. If there are two haplotypes of normal chromosome in breakpoint regions, the embryo will be diagnosed as embryo with normal karyotype. However, if there is one haplotype of rearranged chromosome and one haplotype of normal chromosome, the embryo will be diagnosed as unbalanced embryo. In addition to the CNV analysis described above, the unbalanced rearrangements can also be diagnosed based on haplotype analysis. In order to test for the disease‐causing gene, the haplotypes of 2 Mbp region around the gene were established to determine which one of the carrier's two haplotypes were linked to the normal gene or to the mutant gene. The status of each embryo was evaluated according to the number of mutated gene haplotypes.

### 2.6 Fetus validation

Human chorionic gonadotrophin (hCG) hormone and ultrasound examination after embryo transfer were used to confirm normal intrauterine gestation. PGT results of transferred embryos were confirmed by karyotype analysis and Sanger sequencing of amniotic fluid cells at the second trimester or umbilical cord blood at birth.

## 3 RESULTS

### 3.1 Family history and IVF

**FIGURE 1:**
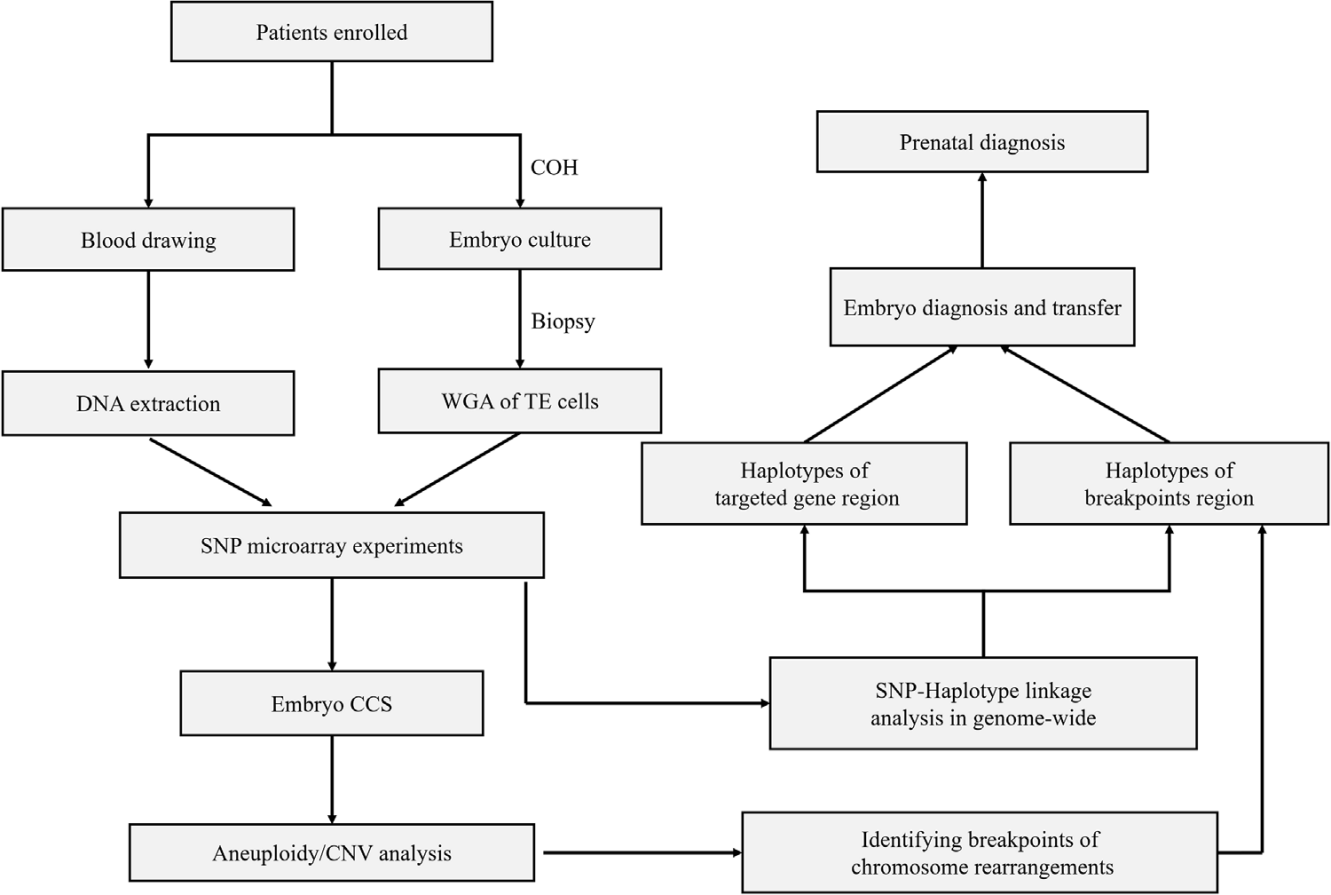
The study designation and workflow. The WGA products of biopsied TE cells and blood DNA of family members were processed with SNP‐array within a single test. Hence, comprehensive chromosome aneuploidy screening was performed in each embryo, the embryos with unbalanced translocation or aneuploidy were identified and excluded from implantation at this stage, and the further CNV analysis of unbalanced embryo could also help to pinpoint the relatively accurate position of breakpoint. Meanwhile, the criterion for informative SNP was defined and family‐based SNP‐haplotype linkage analysis in genome‐wide was performed, and then haplotypes were assigned for the couple, the embryos, and the relatives. The whole genome was divided into large amounts of 2 Mbp windows, which contained sufficient informative SNPs, the positions of the disease‐causing gene and the rearrangement breakpoints would be critically focused on. By linkage analysis, the haplotypes linked to the normal gene/chromosome and to the mutant gene/rearrangement of the carrier couples can be mapped separately. According to the haplotype information in embryos, the status of each embryo with mutant gene and balanced rearrangement can be identified. Detailed haplotype linkage analysis is illustrated in Figures 3 and 4. The unaffected embryos will be transferred into the patients after consulting and prenatal diagnosis of all pregnancies was required. Abbreviation: COH, controlled ovarian hyperstimulation.

**FIGURE 2:**
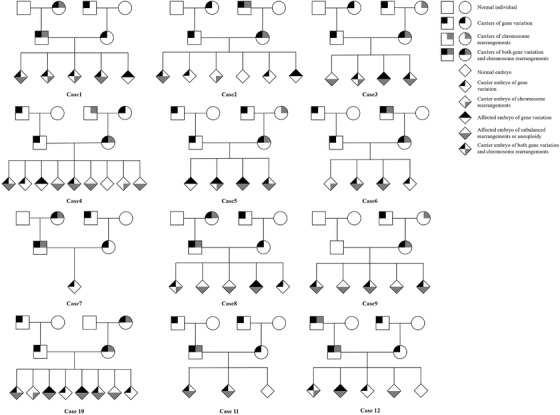
The genetic map of the recruited couples. Twelve families that would undergo IVF were recruited, genetic variants included autosomal recessive (AR) gene, X chromosome‐linked recessive gene, chromosomal‐balanced translocation, and inversion. All gene variants and chromosome rearrangements in couples were respectively inherited from their parents by validation. For all the 59 biopsied embryos, the aneuploidy, disease‐causing gene, and chromosome rearrangements abnormalities were successfully evaluated with one test. Aneuploidy and unbalanced translocation were processed through CNV and haplotype linkage analysis, and gene variants and chromosome rearrangements were conducted by haplotype linkage analysis

In the proposed study, 12 families that would undergo assisted reproductive were recruited, genetic variants included autosomal recessive (AR) gene, X chromosome‐linked recessive gene, chromosomal balanced translocation, and chromosomal inversion. All gene variants and chromosome rearrangements in couples were respectively inherited from their parents, confirmed by validation. Fourteen IVF cycles with PGT were performed, case 2 and case 5 underwent two controlled ovarian hyperstimulation cycles each, and the others had one cycle. Clinical characteristics are shown in Table 1, and the controlled ovarian hyperstimulation results of these patients are listed in Supplementary Table S1. The study designation and workflow is shown in Figure 1 and family pedigree charts of all cases are shown in Figure 2.

**TABLE 1:** The clinical characteristics of patients and embryos transferred in this study

			Cytogenetic location		Gene variant		Rearrangement karyotype	Number of TE	Phasing result	
Patient	Disease	Gene		MI	Male	Female			gene	karyotype
Case‐1	Phenylketonuria	PAH	12q23.2	AR	c.527G > A, mat	c.158G > A, pat	46,XY,t(6;14)(q22;q13), mat	Embryo‐2	Carrier	Carrier
Case‐2	Usher syndrome, type 2A	USH2A	1q41	AR	c.13822C > T, mat	c.8559‐2A > G, pat	46,XX,inv(3)(p26q21), pat	Embryo‐5	Carrier	Normal
Case‐3	Albinism, oculocutaneous	TYR	11q14.3	AR	c.896G > A, mat	c.645G > A, pat	46,XX,t(13;18)(q21;q12), mat	Embryo‐2	Carrier	Carrier
Case‐4	Deafness, Pendred syndrome	SLC26A4	7q22.3	AR	c.2668A > G, pat	c.897G > C, mat	45,XX,rob(14;15)(q10;q10), pat	Embryo‐2	Normal	Normal
Case‐5	Deafness, type 1A	GJB2	13q12.11	AR	c.235del, pat	c.299_300 del, pat	46,XX,t(6;18)(p23;q23), mat	NA	NA	NA
Case‐6	Citrin deficiency	SLC25A13	7q21.3	AR	c.852_855 del, pat	c.2T > C, pat	46,XX,t(4;16)(q25;q13), pat	Embryo‐4	Carrier	Normal
Case‐7	Myasthenic syndrome	DOK7	4p16.3	AR	c.601C > T, mat	c.446C > T, pat	45,XY,rob(13;14)(q10;q10), mat	Embryo‐1^a^	Carrier	Normal
Case‐8	Deafness, type 1A	GJB2	13q12.11	AR	c.235del, mat	c.109G > A, pat	45,XY,rob(13;21)(q10;q10), mat	Embryo‐5	Carrier	Normal
Case‐9	Hemolytic anemia	G6PD	Xq28	XLR	normal	c.1376C > T, mat	46,XX,t(4;10)(q21;q21.2), pat	NA	NA	NA
Case‐10	Deafness, type 1A	GJB2	13q12.11	AR	c.109G > A, pat	c.299_230 del, mat	46,XX,t(4;15)(p15.2;q24), mat	Embryo‐5	Carrier	Normal
Case‐11	Citrin deficiency	SLC25A13	7q21.3	AR	c.852_855 del, pat	c.2T > C, pat	46,XY,t(4;19)(p16.3;q13.42), uk	Embryo‐3	Normal	Normal
Case‐12	Muscular dystrophy	CAPN3	15q15.1	AR	c.589C > T, pat	c.2120A > G pat	45,XY,rob(14;15)(q10;q10), pat	Embryo‐5	Normal	Normal

^a^ This embryo developed into monochorionic monoamniotic (MCMA) twin pregnancy after transfer.

Abbreviations: NA, not available; TE, transferred embryo; uk, unknown.

### 3.2 Linkage analysis by the proposed approach

**FIGURE 3:**
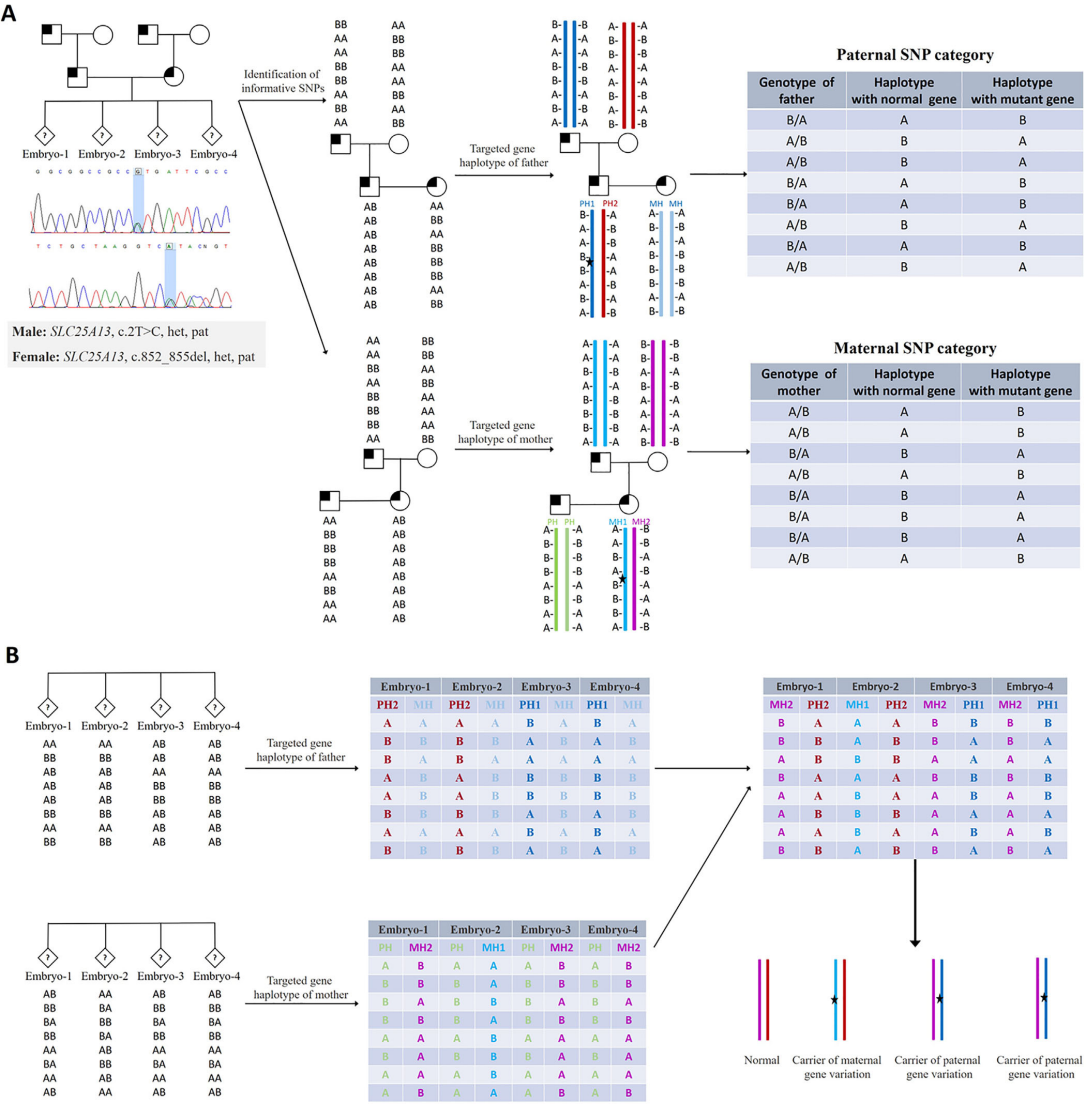
Principles of disease‐causing gene haplotyping. (A) Example of case 6 with an autosomal recessive disorder of Citrin deficiency, the couple was both *SLC25A13* gene variant carriers and inherited from their fathers respectively. DNA from the couple, either of their parents and the WGA products of biopsied TE cells, was genotyped with SNP array and family‐based SNP‐haplotype linkage analysis in genome‐wide was performed by defining informative SNPs, and then the haplotypes linked to the normal gene and to the mutant gene of the carrier couples were identified separately. On the basis of phasing the carrier and his/her parent's haplotype, the recombination events that might occur during meiosis in embryos will be identified clearly. The criterion for informative SNP was that these SNPs should be heterozygous in the carrier and homozygous in his/her partner, in the meanwhile be homozygous in the carrier's parents. The homolog that is inherited from the carrier's father must contain the causative variant and is denoted by H1, whereas homolog H2 that is inherited from the carrier's mother will carrier the normal allele. Subsequently, informative SNPs were categorized to define parental haplotype subcategories—PH1 and PH2 for paternal SNPs and MH1 and MH2 for maternal SNPs. Different colors indicated different haplotype subcategories. (B) Determination of embryo inheritance was based on haplotype information based on informative SNPs. For embryos with both PH1 and MH1 would be diagnosed as affected embryos, for embryos with either PH1 or MH1 would be diagnosed as carrier embryos, and for embryos with neither PH1 nor MH1 would be diagnosed as normal embryos

**FIGURE 4:**
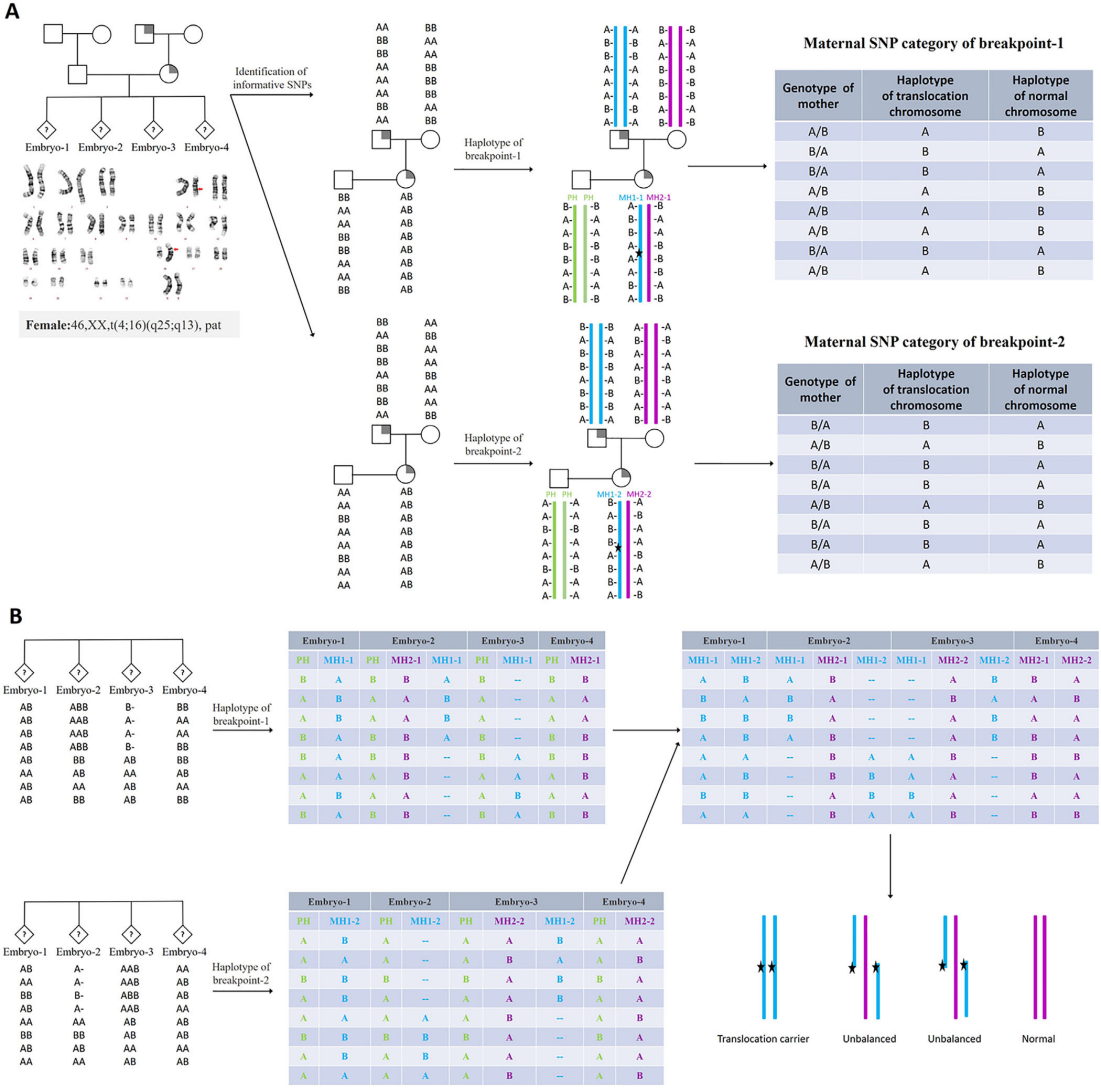
Principles of rearrangement breakpoints haplotyping. (A) Example of case 6 with an translocation of 46,XX,t(4;16)(q25;q13), which was inherited from her fathers. DNA from the couple, either of her parents and the WGA products of biopsied TE cells, was genotyped with SNP‐array methods, and the process of phasing haplotype is similar to Figure 3. The criterion for informative SNP was that these SNPs should be heterozygous in the carrier, homozygous in his/her partner and her parents. The homolog that is inherited from her father must contain the rearranged chromosome and is denoted by H1, whereas homolog H2 that is inherited from her mother will carrier the normal chromosome. As there existed two breakpoints, for the breakpoint in 4q25 region, informative SNPs were categorized to define haplotype subcategories–‐MH1‐1 and MH2‐1, and for the breakpoint in 16q13 region, informative SNPs were categorized to define haplotype subcategories–‐MH1‐2 and MH2‐2. Different colors indicated different haplotype subcategories. (B) Determination of embryo inheritance was based on haplotype information based on informative SNPs. For embryos with either MH1‐1 or MH1‐2 would be diagnosed as unbalanced embryos, for embryos with both MH1‐1 and MH1‐2 would be diagnosed as translocation‐carrying embryos, and for embryos with neither MH1‐1 nor MH1‐2 would be diagnosed as normal embryos. The displayed chromosomes at the bottom right corner represented the two rearranged chromosomes and were not homologous

**FIGURE 5:**
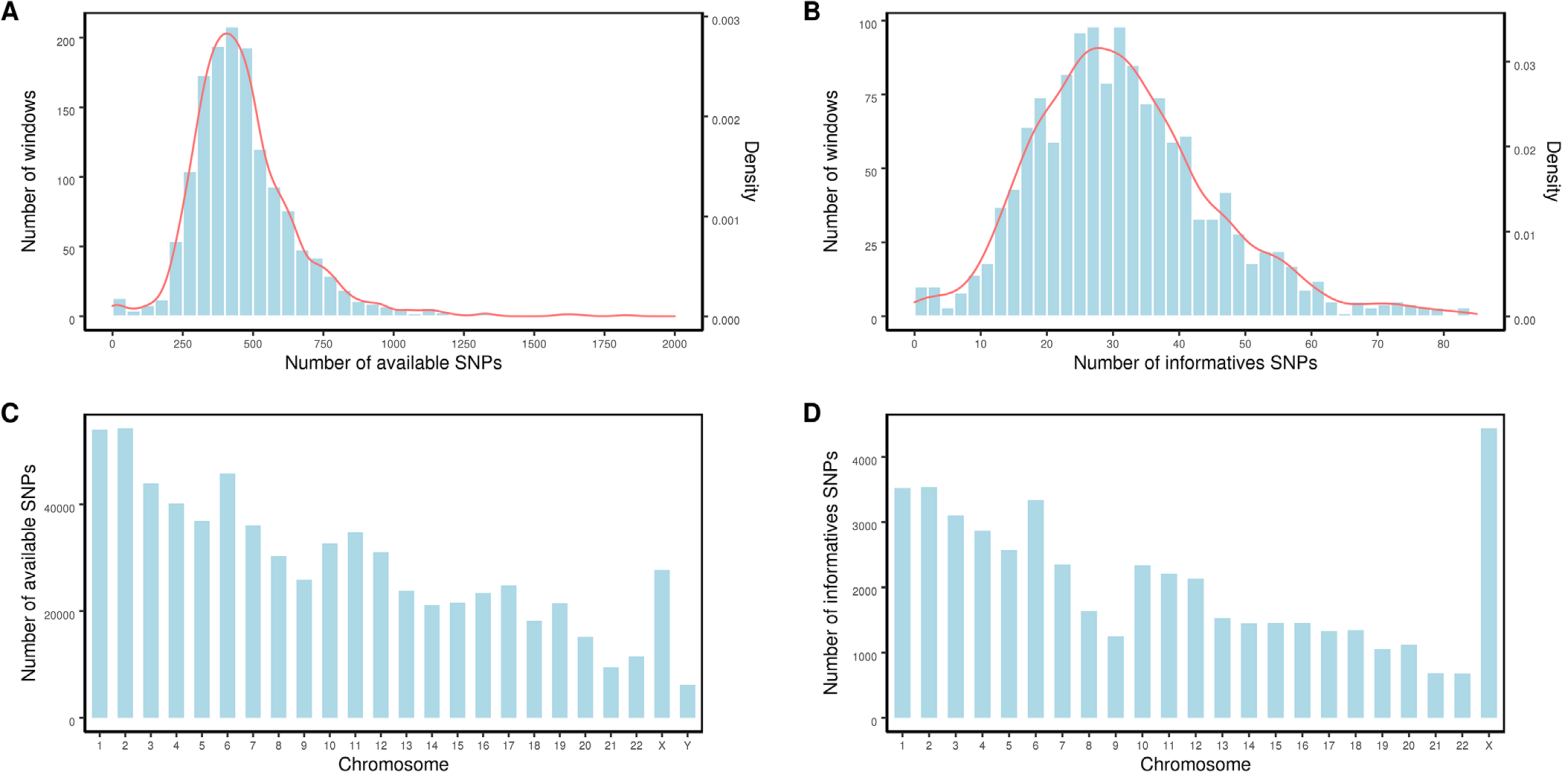
The distribution of available SNPs and informative SNPs in windows or chromosomes. (A) The distribution of SNPs in windows. The horizontal axis represented the number of available SNPs, and the vertical axis represented the number of windows (2 Mb) and density, respectively. (B) The horizontal axis represented the number of informative SNPs, and the vertical axis represented the number of windows (2 Mb) and density respectively. (C) The distribution of SNPs in each chromosome. The horizontal axis represented the number of chromosome, and the vertical axis represented the number of available SNPs. (D) The horizontal axis represented the number of chromosome, and the vertical axis represented the number of informative SNPs. For chromosome Y, all the available SNPs were informative SNPs, so we did not show again in (D)

With our pipeline, haplotypes across the genome were assigned for the couple, the embryos and the carrier's family members. As shown in Figure 1, a few TE cells were biopsied from D5/6 blastocysts and then amplified by WGA. Hence, the amplified products and peripheral blood DNA of the couple and family numbers were processed with SNP‐array. The proposed pipeline was able to establish the haplotypes of any genes or regions covered by the SNP loci in genome‐wide. In this study, the total number of windows was 1561 in the whole genome, some regions with centromeres had no SNP probes distribution, so the final number of windows that contained SNPs was 1476. On average, 98.8% of the windows had two or more informative SNPs and haplotypes could be confidently inferred. The median was about 30 in our study; therefore, one window with 2 Mb had a sufficient number of informative SNPs. Here, the positions of disease‐causing genes and rearrangement breakpoints were critically focused on and used to determine the haplotypes linked to the abnormal variants in the carrier's family, followed by the diagnosis of embryos by theirs haplotypes inherited from the carriers. The molecular karyotype of an unbalanced embryo through CNV analysis could pinpoint the relatively accurate breakpoint position with 100‐200 Kb. If no unbalanced embryo was identified, breakpoint information from the karyotype was used. The procedure of establishing haplotypes and diagnosing embryos free of disease‐causing gene or abnormal karyotypes is shown in Figures 3 and 4. To avoid the false‐negative and false‐positive errors and to increase the reliability of haplotypes, sufficient SNP markers were approached. The distribution of all available SNPs and informative SNPs in windows or chromosomes is shown in Figure 5.

### 3.3 Simultaneous detection of multiple anomalies in one test

For each embryo, the aneuploidy, disease‐causing gene, and chromosomal rearrangements abnormalities were tested with one experiment. Aneuploidies were processed through CNV analysis, whereas gene and chromosome variants were evaluated by family haplotype linkage analysis. The proposed pipeline successfully yielded results of all the 59 biopsied blastocysts, of which 26 were diploid, 24 had rearrangements related abnormalities, and 9 showed *de novo* aneuploidies unrelated to rearrangements. Among the 26 diploid embryos, three were affected by monogenic diseases, seven were free of disease‐causing genes, and the others were carriers of heterozygous recessive variants inherited from father or mother. Further, among these 26 diploid embryos, 15 were with normal karyotypes and the others were carriers of chromosomal structural rearrangements inherited from their parents. The detailed results of the blastocysts tested are shown in Supplementary Table S2. For all embryos diagnosed, the existence of rearranged chromosomes was also successfully predicted by haplotype analysis in addition to CNV analysis, which further demonstrated the accuracy of this proposed approach.

### 3.4 Clinical outcome and confirmation of the efficacy

Twelve blastocysts were thawed, and then transferred into the uterus of female patients. In case 2, case 6, and case 10, the patients achieved pregnancy in the second transfer cycle, while all the other patients became pregnant in their first transfer cycle. In case 7, the embryo developed into a monochorionic monoamniotic twin pregnancy. For all the mothers, karyotype and gene variant analyses of amniotic fluid in the second trimesters or umbilical cord blood at birth were required. We validated that the testing results of biopsied embryonic TE cells and the diagnosis results of fetal cells were consistent, the sensitivity and specificity were both 100%. At the time of manuscript preparation, nine pregnancies had reached the second trimester and five of them had been delivered.

## 4 DISCUSSION

As we know, although researchers have identified causing genes for many diseases, effective treatment is available for only a few and the cost is unbearable for most families; therefore, preventing the birth of affected fetuses is crucial. PGT has now been widely used to select IVF embryos free of disease‐causing genes or chromosomal abnormalities. In the present study, by using family‐based genome‐wide SNP‐haplotype analysis, we developed a universal method that can simultaneously detect monogenic diseases, aneuploidy, and chromosomal rearrangements in a single embryo with one test. The method is applicable for PGT with different indications, such as varieties of monogenic diseases with different genetic inheritances and chromosomal rearrangements with different karyotypes, especially for couples with both disease‐causing genes and chromosome rearrangements.

In the first PGT cycle, the embryos with the risk for an X chromosome‐linked genetic disease were diagnosed by DNA amplification of a Y chromosome specific repeat sequence.^37^ The authors aspirated one or more cells from an embryo cultured and then performed genetic testing, and only the unaffected embryos were transferred with the aim of delivering a newborn without genetic diseases. Over the past decades, genetic techniques have been developed for the diagnosis of a wide range of indications, including single gene disease (PGT‐M), structural rearrangement (PGT‐SR), and chromosome aneuploidy (PGT‐A).^38^ For monogenic diseases, sequencing of PCR products was initially used to directly detect variants in biopsied embryo cells.^12^, ^13^, ^14^ However, owing to the limitations of contamination and ADO,^15^, ^16^ in recent years, family‐based linkage analysis has become a standard method using STR or SNP haplotype analysis to indirectly determine the variants.^17^, ^18^, ^19^, ^20^ Handyside et al developed the Karyomapping method, which could be applied to any single‐gene defect within the regions covered by the SNP loci and overcame the difficulty of ADO at the single‐cell level.^21^, ^39^ Recently, Yan et al reported on the method of “mutated allele revealed by sequencing with aneuploidy and linkage analyses” (MARSALA)^18^ and Backenroth et al developed a 24‐hour all‐in‐one method for PGD of monogenic disorders,^19^ both of which could detect gene variants and chromosome aneuploidies simultaneously in embryos, with the prerequisite being the availability of an affected proband that was used for linkage analysis. Zamaniesteki et al established the method of Haplarithmisis,^22^ which have been proved to be effective methods for clinical PGT‐M. For carriers of chromosomal rearrangements, with the rapid development and application of molecular genetic techniques of CCS, clinical diagnosis and pregnancy rate have been significantly improved.^8^, ^9^, ^10^, ^11^ Hu et al established a method to identify translocation breakpoints by using NGS of a microdissected junction region, and then distinguish karyotypes of embryos by junction spanning PCR and/or linkage analyses.^25^ Moreover, Xu et al reported a method named “Mapping Allele with Resolved Carrier Status” (MaReCs), which enabled the identification of the translocation carrier status in embryos by sequencing in breakpoint region.^26^ Furthermore, some researches applied nanopore sequencing to identify the breakpoint of rearrangements and then evaluated embryo‐carrying status by junction spanning PCR or linkage analysis.^28^, ^40^, ^41^ These published methods cannot only detect the chromosomal imbalances, but can further distinguish embryos with balanced rearrangement karyotypes from those with normal karyotypes,^25^, ^26^, ^27^ preventing the passing on of chromosomal rearrangements to the next generation. However, these methods cannot detect chromosome imbalances/aneuploidies and chromosome rearrangements simultaneously in the same embryo, separate procedures had to be performed. In addition, these methods also required the breakpoint identification of rearrangements to a single base by sequencing, whereas precise rearrangement breakpoints are not essential to predict the chromosome status. Furthermore, monogenic diseases are not applicable. Therefore, it is of great importance to develop one universal PGT technology that is capable of simultaneously testing for aneuploidy, structural rearrangements, and monogenic disorders using a single platform.

In this present study, for inherited variants, the couple and either of their parents could be used for genome‐wide haplotype phasing, our pipeline can obtain the clear haplotypes for each family member, and the haplotypes of the mutant genes and rearrangement breakpoints were determined accordingly. Subsequently, the called SNPs of embryos with the same microarray were pooled into the above pipeline, and genome‐wide haplotypes of embryos were produced and used to indicate the disease status of mutant genes or rearrangements. As shown in Figure 4, the carrier's first genotype is AB, we speculate the carrier's allele A must come from his (abnormal) father, because his variant‐carrying father is A/A homozygous; therefore, the carrier's variant‐carrying haplotype must contain the allele A. Further, we used the similar strategy to deduce the inherited allele of the embryos, the variant‐carrying haplotype would be ABBAAABA and the normal haplotype would be BAABBBAB. This strategy is especially suitable for couples with no affected proband, and one of the carrier's parents or other close relative with known disease status can be used for haplotyping. The exception was when both the husband's parents or both the wife's parents had the same mutation, and himself/herself was a carrier, the method could not be applied, the carrier's siblings might be helpful to phase haplotype. For *de novo* carriers of chromosomal rearrangements, the unbalanced embryos also can be used as a reference. Practically, as the haplotypes across the genome can be established, our strategy is universal for almost all kinds of rearrangements and single‐gene disorders. In addition, the existence of one rearranged chromosome in translocation could also be diagnosed based on haplotype analysis; for embryos with unbalanced translocation, one haplotype of a normal chromosome and another of a rearranged chromosome were presented, which has been proven by CNV analysis, thus further demonstrating the accuracy of the proposed approach. However, one limitation in our research is that an affected proband or embryo is needed for the carrier couples with a *de novo* variant.

Meiosis event is the cellular program underlying gamete formation. For this process, crossovers between homologous chromosomes are a common feature of sexual reproduction and play an essential mechanistic role to ensure regular segregation.^42^ Crossover frequencies vary across different chromosomes within individual nuclei, the average number of crossovers in gametes is about 27 in males^43^ and 50% higher in females,^44^ and occur at a frequency of ∼1% per Mb on a chromosome. In our method, the whole chromosome haplotype of the targeted region and the normal homologous chromosome could be established simultaneously; therefore, the presence of homologous recombination in embryos will be identified clearly, reducing the misdiagnosis rate. Besides, we improved the accuracy of breakpoint locations identified within 200 kb by the molecular karyotype of unbalanced embryos, for which the informative SNPs of 2∼4 Mb flanking the targeted regions were sufficient.

In summary, we have established a comprehensive, practical, and universal PGT strategy, which can detect monogenic diseases, aneuploidy, and balanced chromosomal rearrangements simultaneously in the same embryo with a single test, thus avoiding the inheritance of genetic variants to the next generations. Theoretically, this platform is applicable for patients with a wide variety of monogenic disorders and chromosomal rearrangements, especially for patients with both monogenic disorders and chromosomal rearrangements. Furthermore, our platform does not require a complex experimental procedure; thus, it is be adapted for routine clinical detection in genetic laboratories. The proposed strategy may markedly improve the precision of embryo testing and facilitate the selection of embryos free of genetic diseases through PGT, preventing the birth of affected fetuses.

## CONFLICT OF INTEREST

The authors declare that there is no conflict of interest that could be perceived as prejudicing the impartiality of the research reported.

## ETHICS APPROVAL

Written informed consent was obtained from each family and this study was approved by the Ethics Committee for Human Subject research of the Obstetrics and Gynecology Hospital, Fudan University. We obtained the consent to publish their clinical data from the patients in this study.

## AUTHOR CONTRIBUTIONS

Shuo Zhang, Xiaoxi Sun, and Congjian Xu designed the research and wrote the manuscript; Shuo Zhang, Caixia Lei, Junping Wu, Min Xiao, Jing Zhou, Saijuan Zhu, Jing Fu, Xiaoxi Sun, and Congjian Xu executed the research (Shuo Zhang, Caixia Lei, and Min Xiao performed the microarray analysis; Shuo Zhang, Saijuan Zhu, and Jing Zhou performed the microarray experiments; Jing Zhou performed cytogenetic experiments of amniotic fluid cell and blood. Jing Fu performed the intracytoplasmic sperm injection and blastocyst biopsy experiments; Junping Wu, Xiaoxi Sun, and Caixia Lei collected the cases). Daru Lu, Xiaoxi Sun, and Congjian Xu directed the critical discussion of the manuscript. All authors approved the final manuscript.

## Supporting information

SUPPORTING INFORMATION

SUPPORTING INFORMATION

SUPPORTING INFORMATION

## Data Availability

The data used in the present study are available from the corresponding author on reasonable request.
