# Supplementary figures and images for: A comprehensive and universal approach for embryo testing in patients with different genetic disorders
Source: Clin Transl Med. 2021 Jul 8;11(7):e490. doi: 10.1002/ctm2.490 (PMC8265165; doi:10.1002/ctm2.490)

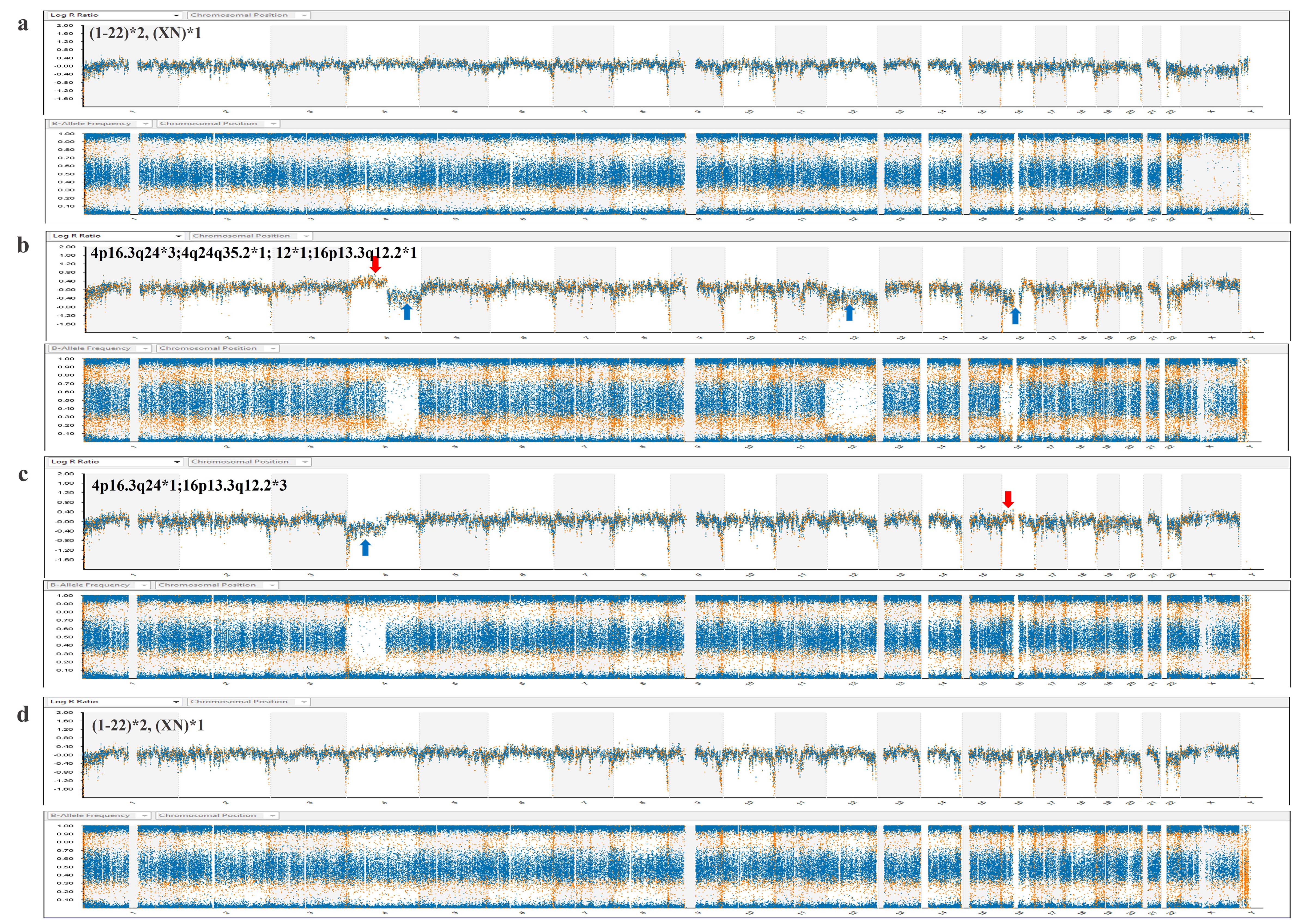

Supplement: Supplementary file 1 — SUPPORTING INFORMATION [file CTM2-11-e490-s003.jpg]
