# Supplementary material for: A comprehensive and universal approach for embryo testing in patients with different genetic disorders
Source: Clin Transl Med. 2021 Jul 8;11(7):e490. doi: 10.1002/ctm2.490 (PMC8265165; doi:10.1002/ctm2.490)
Supplement: Supplementary file 2 — SUPPORTING INFORMATION [file CTM2-11-e490-s002.docx]

| **Supplemental Table S1. The COH characteristics of the patients enrolled in this study** | | | | | | | | |
| --- | --- | --- | --- | --- | --- | --- | --- | --- |
| Case | Maternal age | Karyotype ^a^ | Monogenic diseases | Number of | Number of | Number of | Number of | Number of |
|  | /Paternal age |  |  | oocytes retrieved | MⅡ oocytes | fertilized oocytes | D3 embryos | biopsied blastocysts |
| 1 | 28/30 | 46,XY,t(6;14)(q22;q13), mat | Phenylketonuria | 19 | 15 | 15 | 12 | 5 |
| 2 ^b^ | 27/27 | 46,XX,inv(3)(p26q21), pat | Usher syndrome, type 2A | 24 | 22 | 22 | 19 | 6 |
| 3 | 26/30 | 46,XX,t(13;18)(q21;q12), mat | Albinism, oculocutaneous | 15 | 11 | 11 | 10 | 4 |
| 4 | 27/28 | 45,XX,rob(14;15)(q10;q10), pat | Deafness, Pendred syndrome | 32 | 29 | 26 | 24 | 9 |
| 5 ^b^ | 26/28 | 46,XX,t(6;18)(p23;q23), mat | Deafness, type 1A | 23 | 15 | 15 | 8 | 4 |
| 6 | 36/36 | 46,XX,t(4;16)(q25;q13), pat | Citrin deficiency | 14 | 11 | 11 | 6 | 4 |
| 7 | 31/32 | 45,XY,rob(13;14)(q10;q10), mat | Myasthenic syndrom | 6 | 5 | 5 | 2 | 1 |
| 8 | 28/29 | 45,XY,rob(13;21)(q10;q10), mat | Deafness, type 1A | 7 | 7 | 7 | 6 | 5 |
| 9 | 34/39 | 46,XX,t(4;10)(q21;q21.2), pat | Hemolytic anemia | 21 | 20 | 20 | 15 | 5 |
| 10 | 21/25 | 46,XX,t(4;15)(p15.2;q24), mat | Deafness, type 1A | 36 | 32 | 31 | 16 | 8 |
| 11 | 27/28 | 46,XY,t(4;19)(p16.3;q13.42) uk | Citrin deficiency | 16 | 15 | 12 | 11 | 3 |
| 12 | 30/29 | 45,XY,rob(14;15)(q10;q10), pat | Muscular dystrophy | 10 | 10 | 9 | 9 | 5 |
| ^a^ The karyotypes were identified by peripheral blood cells. | | | | | | | | |
| ^b^ In case 2 and 5, the number of oocytes was from two COH cycles. mat: maternal, pat: paternal, uk: unknown. In case 11, whether the translocation is inherited from parent is unknown | | | | | | | | |
|  | | | | | | | | |
|  | | | | | | | | |
|  | | | | | | | | |
|  | | | | | | | | |
