# Supplementary material for: A comprehensive and universal approach for embryo testing in patients with different genetic disorders
Source: Clin Transl Med. 2021 Jul 8;11(7):e490. doi: 10.1002/ctm2.490 (PMC8265165; doi:10.1002/ctm2.490)
Supplement: Supplementary file 3 — SUPPORTING INFORMATION [file CTM2-11-e490-s001.docx]

**Supplemental table S2. The detailed results of the blastocysts tested** **in this study**

| **Patient** | **Number of** | **Grade of** | **Results of molecular** | **Results of gene** | **Results of chromosome** |
| --- | --- | --- | --- | --- | --- |
|  | **biopsied blastocysts** | **blastocysts** | **karyotype/aneuploidies** | **variants** | **rearrangements** |
| Case 1 | Embryo-1 | 5BC | 6q22.31q27*3;14q12q32.33*1 | Carrier of paternal variation | Unbalanced |
|  | Embryo-2 | 5BB | diploid | Carrier of paternal variation | Carrier |
|  | Embryo-3 | 5BB | diploid | Carrier of maternal variation | Carrier |
|  | Embryo-4 | 5CB | 6q22.31q27*1;14q12q32.33*3 | Carrier of paternal variation | Unbalanced |
|  | Embryo-5 | 5BC | 4q31.21q35.2 | Affected | Normal |
| Case 2 | Embryo-1 | 5BB | 1*3;3p26.3p24.3*1;3q13.33q29*3 | Carrier of maternal variation | Unbalanced |
|  | Embryo-2 | 5BC | diploid | Carrier of maternal variation | Normal |
|  | Embryo-3 | 5BB | diploid | Normal | Carrier |
|  | Embryo-4 | 5BB | diploid | Normal | Normal |
|  | Embryo-5 | 5BB | diploid | Carrier of paternal variation | Normal |
|  | Embryo-6 | 5CB | diploid | Affected | Normal |
| Case 3 | Embryo-1 | 6BC | 13q12.11q21.32*1 | Normal | Unbalanced |
|  | Embryo-2 | 5BC | diploid | Carrier of paternal variation | Carrier |
|  | Embryo-3 | 5BB | 13q12.11q21.33*3;18p11.32q12.1*1 | Affected | Unbalanced |
|  | Embryo-4 | 6BC | 13q21.33q34*3;18q12.1q23*1 | Carrier of maternal variation | Unbalanced |
| Case 4 | Embryo-1 | 5CB | 4*3;19q13.13q13.33*1 | Carrier of maternal variation | Carrier |
|  | Embryo-2 | 5CB | diploid | Carrier of maternal variation | Normal |
|  | Embryo-3 | 5CB | diploid | Affected | Normal |
|  | Embryo-4 | 5BC | 22*1 | Normal | Normal |
|  | Embryo-5 | 5BC | 14*3 | Carrier of maternal variation | Unbalanced |
|  | Embryo-6 | 5CB | 4*3 | Normal | Carrier |
|  | Embryo-7 | 5CB | diploid | Normal | Normal |
|  | Embryo-8 | 5BB | diploid | Normal | Carrier |
|  | Embryo-9 | 5BB | 15*1 | Normal | Normal |
| Case 5 | Embryo-1 | 5BC | diploid | Affected | Normal |
|  | Embryo-2 | 5BC | diploid | Affected | Carrier |
|  | Embryo-3 | 5BB | 6p25.3p22.1*3;18q22.1q23*1 | Affected | Unbalanced |
|  | Embryo-4 | 5BB | 6p25.3p22.1*3;18q22.1q23*1 | Carrier of paternal variation | Unbalanced |
| Case 6 | Embryo-1 | 5BB | diploid | Normal | Carrier |
|  | Embryo-2 | 5BB | 4p16.3q24*3;4q24q35.2*1;12*1;  16p13.3q12.2*1 | Carrier of maternal variation | Unbalanced |
|  | Embryo-3 | 5BB | 4p16.3q24*1;16p13.3q12.2*3 | Carrier of paternal variation | Unbalanced |
|  | Embryo-4 | 5BC | diploid | Carrier of paternal variation | Normal |
| Case 7 | Embryo-1 | 5CB | diploid | Carrier of paternal variation | Normal |
| Case 8 | Embryo-1 | 5BB | diploid | Carrier of paternal variation | Carrier |
|  | Embryo-2 | 5BC | 16*1 | Normal | Normal |
|  | Embryo-3 | 5BC | 15q13.3q26.3*1 | Normal | Normal |
|  | Embryo-4 | 5BC | 6p25.3p21.1*1 | Affected | Carrier |
|  | Embryo-5 | 6BB | diploid | Carrier of maternal variation | Normal |
| Case 9 | Embryo-1 | 5BB | 4q32.1q33*1 | Carrier of maternal variation | Normal |
|  | Embryo-2 | 5BC | 4p16.3q13.3*1;4q32.1q33*3;  10p15.3q11.23*3 | Normal | Unbalanced |
|  | Embryo-3 | 5BC | 4p16.3q13.3*1;10p15.3q11.23*3 | Carrier of maternal variation | Unbalanced |
|  | Embryo-4 | 5BC | 4p16.3q13.3*3;10p15.3q21.1*1 | Normal | Unbalanced |
|  | Embryo-5 | 5BC | 4p16.3q13.3*1;8*1 mos (50%);  10p15.3q21.1*3 | Carrier of maternal variation | Unbalanced |
| Case 10 | Embryo-1 | 5BC | 4p16.3p15.31*1;4p15.31q35.2*3;  15q11.2q25.3*1;15q26.1q26.3*3 | Carrier of paternal variation | Unbalanced |
|  | Embryo-2 | 5BC | diploid | Normal | Carrier |
|  | Embryo-3 | 5BC | 4p15.31q25.2*3;15q11.2q25.3*1 | Affected | Unbalanced |
|  | Embryo-4 | 5BC | diploid | Carrier of maternal variation | Normal |
|  | Embryo-5 | 5BC | 15*3 | Affected | Unbalanced |
|  | Embryo-6 | 5BC | 4p15.31q25.2*3;15q11.2q25.3*1 | Carrier of paternal variation | Unbalanced |
|  | Embryo-7 | 6CB | 4p15.31q25.2*3;15q11.2q25.3*1 | Normal | Unbalanced |
|  | Embryo-8 | 5CB | diploid | Carrier of maternal variation | Normal |
| Case 11 | Embryo-1 | 5CB | 4p16.3*3;19q13.41q13.43*1 | Carrier of paternal variation | Unbalanced |
|  | Embryo-2 | 5CB | 13q22.1q32.31*1 | Carrier of paternal variation | Normal |
|  | Embryo-3 | 5BC | diploid | Normal | Normal |
| Case 12 | Embryo-1 | 5CB | diploid | Carrier of maternal variation | Carrier |
|  | Embryo-2 | 5CB | 14*3 | Affected | Unbalanced |
|  | Embryo-3 | 5CB | diploid | Carrier of maternal variation | Normal |
|  | Embryo-4 | 5BC | 6*3 mos (50%); 15*1 | Normal | Unbalanced |
|  | Embryo-5 | 5BB | diploid | Normal | Normal |
